# Utilization of biochar for remediation of heavy metals in aqueous environments: A review and bibliometric analysis

**DOI:** 10.1016/j.heliyon.2024.e25785

**Published:** 2024-02-07

**Authors:** Zebron Phiri, Nathaniel T. Moja, Thabo T.I. Nkambule, Lueta-Ann de Kock

**Affiliations:** Institute for Nanotechnology and Water Sustainability (iNanoWS), College of Science Engineering and Technology, University of South Africa, Florida Science Campus, Johannesburg, 1710, South Africa

**Keywords:** Biochar, Bibliometric analysis, Heavy metals, Adsorption, Bibliometrix, Wastewater, Meta-analysis

## Abstract

Biochar usage for removing heavy metals from aqueous environments has emerged as a promising research area with significant environmental and economic benefits. Using the PICO approach, the research question aimed to explore using biochar to remove heavy metals from aqueous media. We merged the data from Scopus and the Web of Science Core Collection databases to acquire a comprehensive perspective of the subject. The PRISMA guidelines were applied to establish the search parameters, identify the appropriate articles, and collect the bibliographic information from the publications between 2010 and 2022. The bibliometric analysis showed that biochar-based heavy metal remediation is a research field with increasing scholarly attention. The removal of Cr(VI), Pb(II), Cd(II), and Cu(II) was the most studied among the heavy metals. We identified five main clusters centered on adsorption, water treatment, adsorption models, analytical techniques, and hydrothermal carbonization by performing keyword co-occurrence analysis. Trending topics include biochar reusability, modification, acid mine drainage (AMD), wastewater treatment, and hydrochar. The reutilization of heavy metal-loaded spent biochar includes transforming it into electrodes for supercapacitors or stable catalyst materials. This study provides a comprehensive overview of biochar-based heavy metal remediation in aquatic environments and highlights knowledge gaps and future research directions.

## 1 Introduction

The contamination of water bodies by heavy metals has become a growing global concern due to the detrimental effects of these pollutants on the environment and human health. Heavy metals are naturally occurring metals with an atomic number of over 20 and an elemental density exceeding 5 g⋅cm^−3^ [1], [2]. Various anthropogenic activities, including industrial processes, agriculture and forestry, mining operations, fossil fuel combustion, and waste disposal, release heavy metals into water sources. These activities result in heavy metal pollutants entering water sources through runoff or underground leaching [3], [4].

Mining operations uncover minerals such as pyrite, marcasite, arsenopyrite, chalcopyrite, sphalerite, and galena to oxidizing agents, producing acid mine drainage (AMD) in the process [5], [6]. Typical AMD is highly acidic, rich in metals, metalloids, and sulfate ions emanating from active, abandoned, or reclaimed mines [7], [8]. The agriculture sector's highest contributors to heavy metal pollution are fertilizers and pesticides [3], [9]. Some industrial processes contributing to heavy metal contamination are smelting, electroplating, automobile batteries, and paints. The heavy metals also come from the waste disposed from these processes [10], [11], [12]. The toxic nature of heavy metals poses severe consequences for aquatic life, animals, and human health, as they can accumulate in the food chain and persist for extended periods [13], [14]. The inability of the body to metabolize heavy metals leads to their bioaccumulation in soft tissues, causing dysfunction in vital organs such as the brain, liver, heart, kidneys, and other critical systems [15], [16], [17]. Consequently, the remediation of heavy metals from aqueous environments is essential for protecting the environment and human health.

While various wastewater treatment techniques for heavy metal remediation exist, such as chemical precipitation [18], reverse osmosis [19] coagulation [20], membrane separation [21], ion exchange [22], electrodialysis [23], and electro-deionization [24], they often require substantial chemical inputs or energy consumption and may not be highly efficient. Adsorption has emerged as a favorable alternative for eliminating heavy metals from aqueous media due to its operational flexibility, cost-effectiveness, and high efficiency [25], [26]. Among the adsorbents investigated recently, biochar has shown potential for heavy metal removal due to its versatile physical and chemical characteristics [27], [28]. Biochar is an aromatic ring-rich structure generated through biomass carbonization under limited oxygen or an inert environment [29] using conventional pyrolysis, microwave-assisted pyrolysis, hydrothermal carbonization, or gasification [30], [31]. Biochar offers a low-cost, renewable, and sustainable solution for various environmental challenges, including heavy metal remediation in wastewater treatment, soil improvement, carbon sequestration, energy production, and as a potential catalyst precursor in diverse applications [30], [31], [32].

Biochar has a stable carbon structure and exhibits strong adsorption properties resulting from its high pore volume, specific surface area, carbon content, and abundant functional groups. These properties make it an effective adsorbent for heavy metals removal [33], [34]. It can simultaneously immobilize multiple heavy metals from aqueous media, making it a versatile adsorbent [35], [36]. The utilization of biochar and its modified form has been on the rise with aspirations of providing a material that is efficient in the remediation of heavy metals from aqueous environments and beyond [37], [38], [39], [40]. However, a comprehensive perspective on using biochar for remediating heavy metals in aquatic environments is still needed; the current knowledge has gaps and limitations.

To gain comprehensive insights and identify knowledge gaps, an extensive bibliometric analysis must be performed to provide a broad understanding of the application of biochar in remediating heavy metals within aquatic systems. Bibliometrics, a term introduced by Pritchard, is a powerful technique to measure scholarly literature and reveal how specific research areas evolve [41], [42]. Bibliometric analysis uses quantitative methods to identify trends and challenges in research [43], [44]. It analyzes publication and citation data [41], [45] to provide valuable insights into publication features such as authors, sources, institutions, journals, citations, and co-citation networks [42].

One of the challenges of bibliometric analysis is how to present and communicate the results to different audiences effectively. Data visualization is a powerful technique that can help to overcome this challenge by transforming complex and large-scale bibliometric data into intuitive and interactive graphical representations [46]. Visualization can enhance the understanding and interpretation of bibliometric analysis by revealing patterns, trends, clusters, gaps, outliers, and relationships that might otherwise be difficult to detect or comprehend [47]. Various types of bibliometric networks can be visualized, such as citation networks, bibliographic coupling networks, and keyword co-occurrence networks [48]. Each type of network represents a different aspect of the scientific literature and can be used to address different research questions or objectives [46].

Bibliometric analysis software tools like Bibliometrix [49], [50], VOSviewer [51], [46], and CitNetExplorer [52] enhance this analytical capability by enabling efficient visualization and analysis of bibliographic data. Researchers from various disciplines leverage bibliometrics to improve performance assessments and better understand research trends [53]. For instance, Prabakusuma and co-workers [54] performed a bibliometric analysis on the use of agri-food wastes and by-products as self-sufficient fish feed materials. They visualized the profiles of selected fields using VOSviewer and Biblioshiny (a web-based interface of Bibliometrix). Wu et al. [55] holistically reviewed biochar research in 2020 using bibliometric analysis to determine critical topics associated with biochar and research trends. Using various methods and indicators, Abdeljaoued et al. [56] conducted a bibliometric study to examine the trends and outputs of biochar research from 2005 to 2019. The study focused on biochar production and valorization pathways, which were assessed based on publications recorded in the Web of Science database.

This study aims to offer valuable insights and highlight trends in the use of biochar for heavy metal remediation in aquatic environments. In particular, this study explores the potential of biochar as a sustainable solution for heavy metal contamination in aquatic systems. Additionally, the study reveals the current knowledge and research trends in this field by conducting a thorough bibliometric analysis and literature review. The study also examines biochar production, modification, adsorption mechanisms, environmental impact, and reusability. Furthermore, the study identifies the existing research gaps and suggests areas for future investigation.

## 2 Bibliometric analysis

We used bibliometric analysis to identify research trends and literature gaps and determine a foundation for future research [57]. By applying the PICO (Population, Intervention, Comparison, Outcome) framework, the research question focused on evaluating the application of biochar as an adsorbent for mitigating heavy metal contamination in water systems.

### 2.1 Data collection and analysis

**Figure 1:**
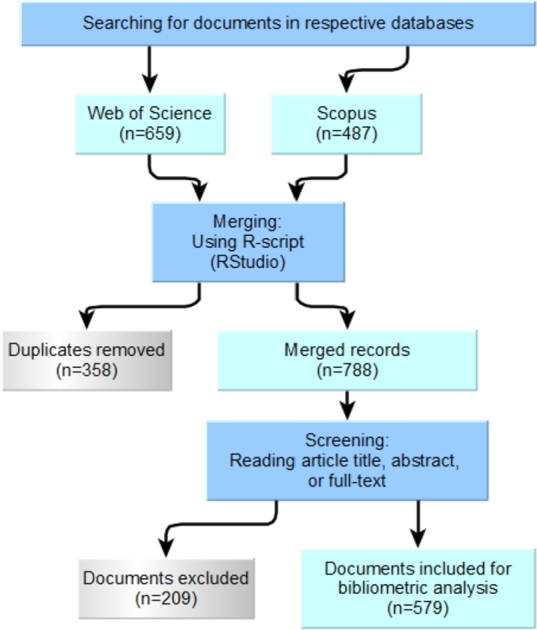
A PRISMA approach used for preliminary data processing.

To find relevant literature, we searched Scopus and Web of Science (WoS) Core Collection databases. Scopus and WoS are the two largest global science databases. We used the preferred reporting items for systematic reviews and meta-analyses (PRISMA) method proposed by Moher et al. [58] to retrieve and process information from Scopus and WoS databases for bibliometric analysis. Fig. 1 depicts the sequential stages of applying the PRISMA data processing methodology. The PRISMA approach is a widely recognized and effective tool for systematic review and meta-analysis of studies. The steps involved in this approach, as shown in Fig. 1, are structured and rigorous, ensuring comprehensive and unbiased data analysis.

We searched the respective databases using the core keywords “biochar” and “heavy metals” and their relevant synonymous terms, as commonly used in the field. We applied wildcards and operators to find relevant terms and variations, ensuring the accuracy of the data processed from the respective databases. Biochar is synonymous with “biochar*,” “bio char*” or “charcoal*”. At the same time, heavy metals are associated with “metal ion*,” “toxic metal*,” “toxic heavy metal*,” “metal ion*,” “* mine drainage,” “acid metal* drainage” as well as “wastewater” or “waste water.” We applied several filters to exclude irrelevant articles such as conference papers, review articles, books, and book chapters, as they are less likely to contain novel information as they might have already reported on existing knowledge [59]. The searches were confined to English research articles published between 2010 and 2022, as they likely contain original contributions. We accessed the respective databases on January 24, 2023.

In the present investigation, we aimed to examine biochar's usage in the remediation of heavy metals from aquatic systems. We excluded articles that primarily addressed soil enhancement, removal of organic pollutants (dyes, pesticides, pharmaceuticals, and the like), bioenergy generation, and carbon capture, as these topics have also garnered considerable interest among researchers working on biochar [55]. Scopus yielded 487 document entries, while WoS generated 659 documents. We downloaded the full records of these articles in bib (Scopus) and plain text (WoS) format. We merged the respective records from Scopus and WoS databases using a script in R (RStudio® version 4.2.2), removed 358 duplicated records and generated an integrated dataset with 788 document entries. We have provided the R code (script) applied in the Supplementary material (see Data availability). Echchakoui [60] demonstrated that bibliometric analysis using solely Scopus or WoS falls short of offering a comprehensive perspective on the knowledge. Caputo and co-workers [43] echoed the same argument and performed bibliometric analysis by merging bibliographic metadata from the Scopus and WoS databases using Bibliometrix. We preliminary examined the titles and abstracts (or full-text) of the respective documents in the merged dataset to eliminate articles beyond the purview of the envisaged bibliometric analysis. As a result, we retained 579 document entries for subsequent analysis.

The 579 acquired documents were subjected to analysis using Bibliometrix (version 4.1), an R-based tool. The analyses employed science mapping, a technique that utilizes bibliometric tools to determine trends in scientific research [49]. It provides compelling evidence of theoretically defined categories in articles while adding quantitative precision to subjective literature evaluation [61]. Various indicators were analyzed to gain insight into heavy metals removal by biochar. These indicators included an overview section encompassing the primary information, annual scientific production, and a three-field plot. Sources were also analyzed; they encompass the most relevant sources and source dynamics. We also elucidated the most contributing institutions and countries, the scientific production of each country, and the intensity of collaborations between countries. Furthermore, an analysis of information extracted from the main body of documents, including the most frequent words (word cloud), trendy topics, and the conceptual structure, such as a co-occurrence network, were conducted.

### 2.2 Data visualization

**Figure 2:**
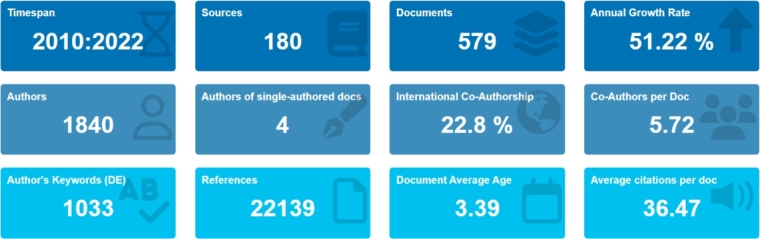
A summary of primary information obtained from data analysis.

We used Biblioshiny, a web-based interface for Bibliometrix, to produce the data for creating visualizations [49], [50] unless we explicitly specified otherwise. Fig. 2 illustrates a snippet from Biblioshiny, which concisely summarizes the primary information extracted from the merged database. The presented data furnishes an overview of key parameters, including but not limited to the annual growth rate, the duration of the considered period, the number of accumulated documents, and the contributing sources.

**Figure 3:**
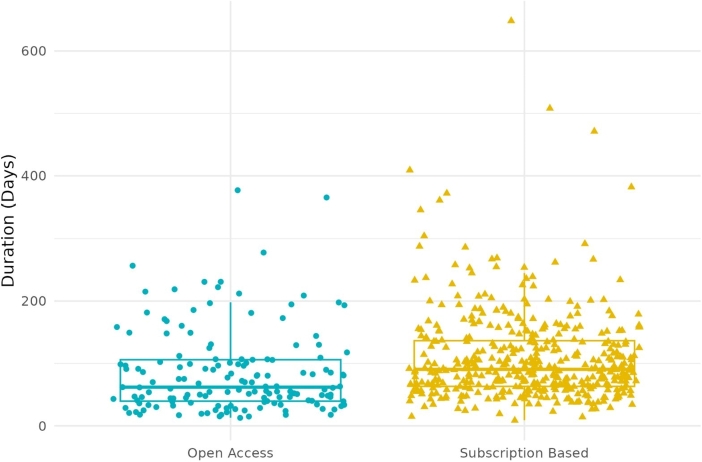
Duration from submitting to acceptance for open-access and subscription-based articles.

In our analysis of 579 articles, 26.42% were open-access. We excluded six articles (two open-access and four subscription-based) that did not have article history information from the analysis of publication frequency. This analysis measured how long it took for an article to be published after being submitted. The distributions were skewed to the right (see Supplementary material under Data availability), so we used the Mann-Whitney U test (Wilcoxon rank test) [62] to compare the time it took for an article to be published for open-access and subscription-based. The test showed a significant difference (W= 22756, p = 1.835e-07) between the Journals. Open-access articles had a median publication time of 62.0 days, while subscription-based articles had a median duration of 90.5 days. We used a box plot to compare the time to publication of open-access and subscription-based articles, as shown in Fig. 3.

#### 2.2.1 Annual scientific production

**Figure 4:**
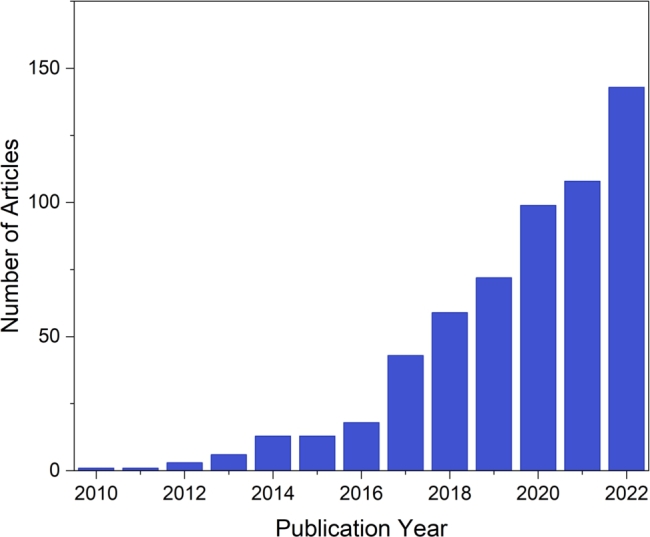
Annual global scientific production of research articles.

Based on an extensive data analysis and visualization of 579 documents authored by 1840 scholars across 180 journals published from 2010 to 2022, it is apparent that the research activities on the usage of biochar for heavy metals remediation began to gain momentum in 2012, as illustrated in Fig. 4. It has consistently exhibited an upward trend with an annual growth rate of 51.22%. The growing recognition of biochar influences this notable trend as an effective and sustainable remediation strategy and an increasing demand for ecologically friendly and sustainable solutions to environmental challenges. The trend emphasizes this focus area's marked expansion and increasing prominence over the examined period.

#### 2.2.2 Country scientific production

**Figure 5:**
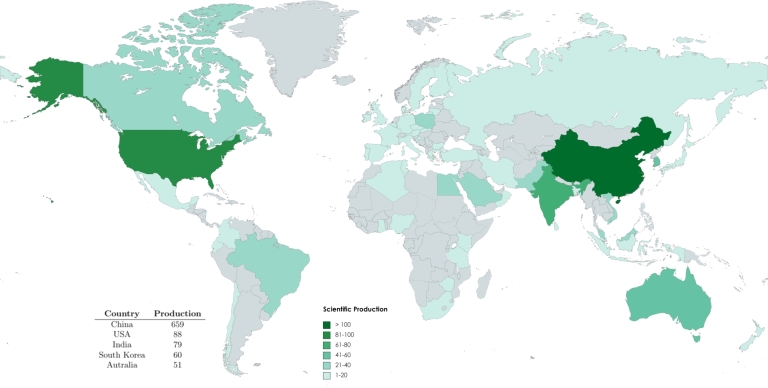
Scientific production by country.

Fig. 5 displays the scientific production of the countries. The world map was generated through MapChart using data from Biblioshiny. Various shades of green denote the representation of global scientific production ranges, each indicating a distinct level of productivity. The accompanying scale in Fig. 5 displays the range of each shade, with dark green signifying a high level of productivity exceeding 100 articles and grey representing a complete absence of articles. The field involving heavy metals reduction by biochar in aqueous media has experienced a significant increase in research activities globally over the past decade. Bibliometric analysis from 2010 to 2022 reveals that China leads the pack with 659 research articles, followed by the United States with 88 articles. India, South Korea, and Australia complete the top five with 79, 60, and 51 articles, respectively. These figures highlight the intense research interest in this area and indicate China's dominance in the global scientific community concerning heavy metals reduction by biochar in aquatic environments.

According to Bibliometrix [49], the country scientific production metric determines the number of “author appearances by country affiliations.” In essence, this implies that for a given article, if three authors belonging to China, the United States, and India have contributed, then the count of appearances for each of these three countries will be increased by 1. This procedure enables tracking research output and impact across different countries and provides valuable insights into the global scientific landscape. Additionally, this measure is a common tool for identifying research patterns and international collaborations, ultimately leading to the exchange of information and advancements in the scientific community on a global scale.

#### 2.2.3 Three-field plot

**Figure 6:**
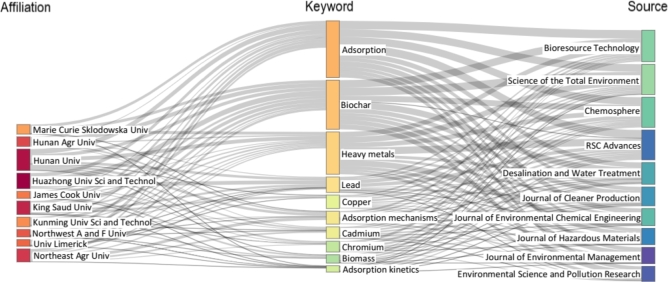
A Sankey diagram showing the respective proportion of research topics by affiliation and publication source.

Fig. 6 details the relationship between research topics, affiliations, and publication sources using a three-field plot, also known as a Sankey diagram [63]. The diagram effectively showcases the proportion of research topics for each affiliation and the corresponding journals they use to publish their findings.

The research topics that form the core of the research area are adsorption, biochar, and heavy metals. These topics are pivotal in advancing knowledge in the field of environmental science and have received a considerable amount of attention from researchers. The most prominent institutions contributing to research in this area include Hunan University, Huazhong University of Science and Technology, King Saud University, and Northeast Agricultural University. These institutions have made significant contributions to the field, and their research output is well-respected by peers and industry experts.

The study found that Bioresource Technology, Science of The Total Environment, and Chemosphere are the preferred journals for publishing research in this area. These journals are highly regarded and are known for publishing high-quality research that advances knowledge and understanding of environmental science. This information can guide researchers on which journals are best suited to their research interests and increase the visibility and impact of their work.

The plot in Fig. 6 provides a comprehensive overview of the distribution of research topics among different institutions and the journals they choose to publish in, providing valuable insights into the trends and patterns of research in this field. The findings can inform future research efforts and collaborations, ultimately leading to advancements in the field and the development of sustainable solutions to address environmental challenges.

#### 2.2.4 Sources dynamic production

**Figure 7:**
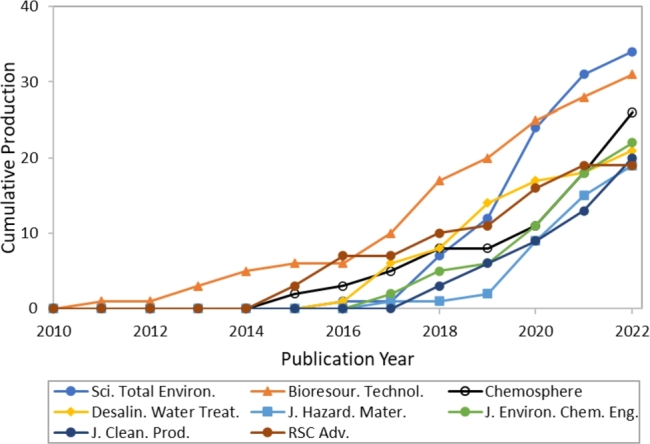
Distribution of literature sources on an annual basis.

Fig. 7 serves as a visual representation of the cumulative production of sources over time. It highlights the evolution and development of sources. Bibliometric analysis of research articles published between 2010 to 2022 on heavy metals remediation by biochar has revealed that the Science of The Total Environment, Bioresource Technology, and Chemosphere are the top contributing sources. The Journal of Environmental Chemical Engineering, Desalination, and Water Treatment, Journal of Cleaner Production, Journal of Hazardous Materials, and RSC Advances also emerged as significant contributors. These findings suggest that heavy metals remediation by biochar is a highly researched area of environmental science and engineering. Researchers and practitioners can draw upon the vast knowledge base from these sources to develop effective strategies for heavy metals removal from contaminated water. The information from these sources can also inform policies and regulations related to heavy metals remediation.

**Figure 8:**
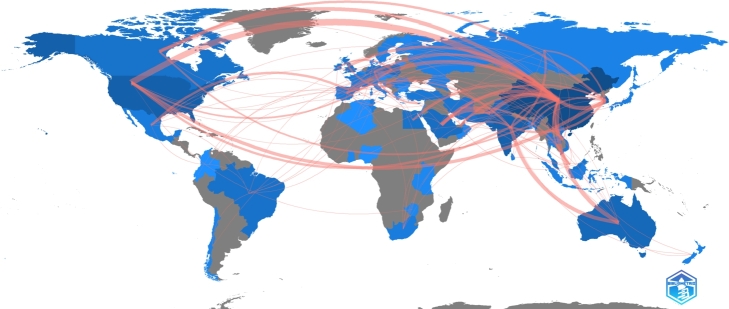
The global collaboration network among countries for the remediation of heavy metals using biochar.

The study utilized Bibliometrix software to conduct network analysis and visualize the collaboration network of countries, as illustrated in Fig. 8. The analysis demonstrated that using biochar for heavy metals remediation is a globally recognized research field, with contributions from diverse countries. The collaboration map affirms that China was the leading contributor to the field, with significant collaborations with the United States, South Korea, Pakistan, and Australia. The study also highlights strong collaborations between countries within the same region, such as Australia and India, and between neighboring countries, such as Pakistan and Saudi Arabia. The network analysis highlights the importance of collaboration among countries to address the global challenges of heavy metal pollution and can assist researchers seeking to establish collaboration networks.

#### 2.2.5 Word cloud

**Figure 9:**
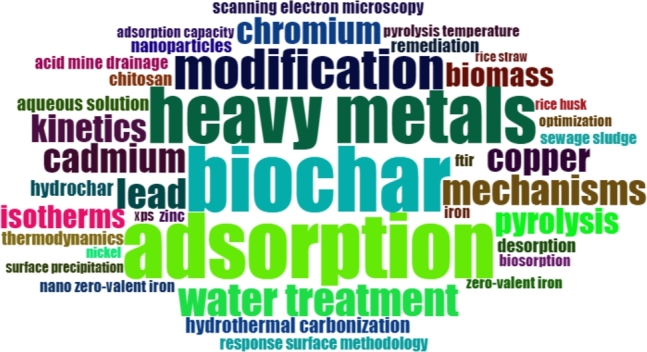
A word cloud of author keywords based on the interaction of heavy metals with biochar in aquatic environments.

Academic papers commonly employ keywords to classify their core research and subject matter. Such keywords provide a concise summary of the primary content of the paper and serve as valuable tools for characterizing its contents and the underlying research direction. In order to generate an accurate representation of the most frequently occurring keywords, a threshold of at least five instances of each term was established for inclusion in the word cloud analysis [64]. Fig. 9 depicts the resulting word cloud visualization, created using the square root of the frequency of each populated keyword.

This approach allows for a more balanced representation of the most prominent terms, enhancing the overall interpretability and usefulness of the generated visualization. These keywords reflect the current research focus in the field, with a particular emphasis on developing novel solutions for water treatment, utilizing materials such as biochar and modified biochar for the effective adsorption of heavy metals. Based on the analysis of the word cloud, it is evident that the adsorption process is the predominant method for removing heavy metals from aquatic systems using biochar.

Including noteworthy terms such as “water treatment” and “biochar modification” underscores the significant process involved and the researchers' focus on enhancing the effectiveness of biochar through modification. Furthermore, the heavy metal ions that have received the most extensive investigation are cadmium, chromium, lead, and copper. We found a significant number of keywords that indicate that the research on using biochar for heavy metal remediation in aquatic environments is a multi-faceted niche with broad perspectives.

#### 2.2.6 Keyword co-occurrence analysis

**Figure 10:**
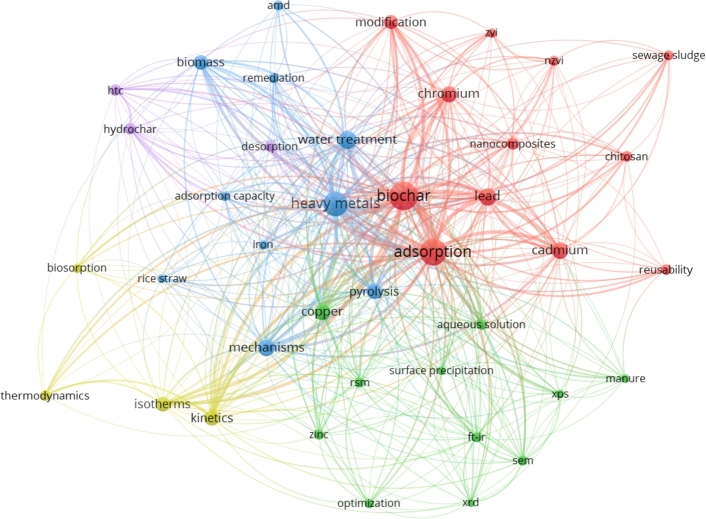
Keyword co-occurrence network based on interaction of heavy metals with biochar in aquatic environments (Created using VOSviewer version 1.6.19).

Word clouds illustrate the frequency of words in a given text pool but do not convey the relationships between the words or provide contextual information. To address this shortcoming, keyword co-occurrence network visualization, as depicted in Fig. 10, can resolve this issue effectively. Clustering analysis allowed us to display the relationship between keywords and directly observe their correlation, thereby facilitating the identification of research areas that are particularly relevant or widely studied within the field [44], [65]. The proximity of keywords to one another reflects their similarity, with those positioned closer together forming a tighter cluster, while those situated farther apart will branch off into distinct groupings [66].

We utilized the co-occurrence technique to examine the relationship between author keywords in the data set. The counting method implemented was frequency, which allowed for determining the most commonly occurring keywords and the strength of their associations. The unit of analysis applied was author keywords, which authors provide to describe the main topics covered in their research. We required a minimum of 5 occurrences of each keyword to ensure meaningful results for inclusion in the analysis. This approach enabled the identification of key themes and patterns in the data set and provided insights into the research interests and priorities of the authors. These techniques allowed for a rigorous and comprehensive analysis of the authors' keywords in the data set.

In essence, the visual representation of the keyword network involves several elements. Firstly, the node size conveys the frequency of occurrence of the keyword, which is the number of times it appears. Secondly, the link between the nodes indicates the co-occurrence of the keywords or the instances where they appear together. Thirdly, the thickness of the link denotes the frequency of co-occurrence or the number of times the keywords appear together. Additionally, the node size correlates with the frequency of the keyword, while the link thickness is proportional to the frequency of co-occurrence. Finally, using different colors serves to identify thematic clusters, which can be interpreted based on the topics covered by the nodes and the relationships between them manifested by the links [44].

Utilizing keyword co-occurrence analysis has the potential to anticipate forthcoming research endeavors in a given field. This visualization can be achieved by incorporating significant “words” from the implications and projected directions of future research presented in publications into the analysis [44]. The findings from keywords co-occurrence analysis conducted using VOSviewer [51] indicate that the primary way in which biochar interacts with heavy metals in aqueous media is through adsorption. Moreover, the clustering analysis revealed four main research directions in this field. These directions encompass the following: (1) the application of characterization techniques; (2) adsorption – the heavy metals removal by biochar modified with nanoparticles or additives such as chitosan; (3) models – the application of modeling techniques, including kinetics, isotherms, and thermodynamics; and (4) water treatment – mechanisms of heavy metals during water treatment or remediation of wastewater, such as acid mine drainage (AMD). An emerging fifth cluster centered around hydrothermal carbonization (HTC) and its product hydrochar is also visible.

Visualizing keyword co-occurrence clusters reveals that biochar modification is crucial in heavy metals remediation. In recent years, researchers have focused on including nanomaterials in biochar matrices to form biochar nanocomposites with enhanced adsorption capacity for heavy metals [67], [68]. Among the nanoparticles, the incorporation of nano zero-valent iron (nZVI) has gained significant attention due to its high reactivity and strong affinity towards heavy metals [69]. The integration of nZVI into biochar has been shown to enhance its adsorption capacity for heavy metals, making it a prospective material for remediation [15], [70].

Another prominent choice for biochar modification among researchers is the usage of chitosan, a biopolymer derived from deacetylating chitin [71]. Chitosan has many amino and hydroxyl groups on its surface, making it an ideal material for adsorption and forming stable chelates with heavy metals [72]. Therefore, researchers have found that chitosan enhances the surface functionality of biochar, increasing its adsorption capacity for heavy metals. Chitosan-modified biochar has been shown to have a higher selectivity for heavy metals, making it an attractive material for heavy metals remediation [73]. Chitosan is widely recognized as an excellent material for adsorption due to its low cost, abundance, non-toxicity, biodegradability, natural antibacterial properties, and lack of secondary pollution [74].

The primary cluster (adsorption) also highlights the extensive research of heavy metals, chromium, and lead using biochar. While biochar has shown promising results in removing these heavy metals, other metals, such as copper and iron, be present in a separate cluster of co-occurring keywords. This occurrence is most likely due to the frequent use of copper and iron as dopants in biochar for wastewater treatment. Although research on the remediation of iron using biochar is not prevalent, the use of dopants such as copper and iron (e.g., ZVI, nZVI, and Fe_3_O_4_) has shown promising results in enhancing the sorption capacity of biochar for heavy metals [75]. Therefore, further exploration of the use of these metals as dopants in biochar is warranted to enhance their effectiveness.

Another cluster highlights the usage of hydrochar, which is essentially biochar derived from hydrothermal carbonization. Pyrolysis temperature significantly impacts adsorption capacity in most cases, requiring considerable energy input. On the other hand, biomass hydrothermal carbonization can be conducted at relatively low temperatures (180–260 °C) [76].

The concept of “reusability” in the context of heavy metal-laden spent biochar entails the potential to reuse, regenerate, and recycle the material. This aspect of the research field has generated considerable interest among researchers who seek to mitigate secondary pollution caused by the leaching or desorption of heavy metals. This endeavor aims to meet circular economy requirements, using spent biochar to create valuable products or benign byproducts.

Many researchers have employed modeling techniques, including isotherms, kinetics, and thermodynamics, to assess the effectiveness of removing heavy metals from synthetic or actual industrial wastewater through batch and columns experimental set-ups or constructed wetlands. Analytical techniques encompassing FTIR, SEM, XRD, and XPS are widely used for biochar characterization and other involved materials [75]. The utilization of response surface methodology (RSM) to optimize experimental work on heavy metal removal by biochar is also evident from the co-occurrence visualization, implying that many researchers have applied this resourceful technique.

#### 2.2.7 Trend topics

**Figure 11:**
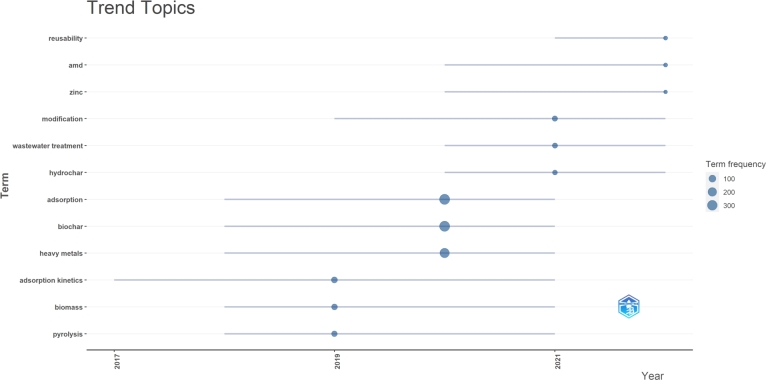
Trending topics on heavy metals removal by biochar.

An extract from Biblioshiny in Fig. 11 displays the progression of trending topics, providing valuable insights into the most recent research activities (or direction) between 2020 and 2022. Based on the collected literature, the identified trending topics were “reusability,” “modification,” “acid mine drainage (AMD),” “wastewater treatment,” “zinc,” and “hydrochar.”

This analysis suggests that the primary focus of a substantial number of researchers working on wastewater treatment is still centered on the search for efficient biochar through modification. The visualization of keyword co-occurrence clusters (Fig. 10) has also drawn attention to the crucial role that biochar modification plays in heavy metal remediation.

AMD is a significant global environmental challenge due to its high levels of toxic heavy metals has taken priority for nations and organizations seeking solutions by using biochar for remediation [77], [78]. Biochar is an effective adsorbent for the removal of heavy metals from AMD. The properties of biochar, such as its high surface area and pore volume, as well as its ability to neutralize acid-impacted water [79], [80], make it an attractive option for treating AMD. Studies have also shown that biochar can be combined with other treatment methods, such as lime neutralization and microbial treatment, to further enhance the removal efficiency of heavy metals from AMD [80], [81].

The keyword co-occurrence visualization also drew attention to the growing interest in exploring the potential of hydrochar. Hydrochar attributes appeal to many and highlight effective heavy metal mitigation in wastewater treatment. Zinc is often mentioned in literature as a heavy metal found in water systems [82]. Additionally, ZnO nanoparticles and ZnCl_2_ have been effectively utilized to create modified biochar that shows significant improvement in removing heavy metals [83], [84].

#### 2.2.8 Knowledge gaps

The research conducted on the efficacy of biochar in mitigating heavy metal ions such as chromium (Cr), lead (Pb), cadmium (Cd), and copper (Cu) has been remarkable. Nevertheless, the exploration of biochar's potential for eliminating various metals (and metalloids) like mercury (Hg), nickel (Ni), zinc (Zn), silver (Ag), iron (Fe), manganese (Mn), molybdenum (Mo), antimony (Sb), cobalt (Co), and others, remains relatively limited in current research. It is crucial to expand the knowledge base and increase investigations of the behavior of other heavy metal ions on biochar. It should be emphasized that most research has relied on synthetic effluent water that contains particular heavy metal ions. Therefore, it is paramount to conduct investigations using heavy metal-laden industrial effluents, such as AMD, to determine the performance of biochar in real-world scenarios.

Researchers have conducted considerable investigations on this topic, but most of it has been limited to bench-scale experiments. Only a few pilot-scale investigations and field studies, such as the use of constructed wetlands for AMD remediation, have been performed. To fully comprehend the efficacy of biochar in industrial contexts, it is imperative to conduct tests employing pilot-scale or industrial equipment. Henceforth, apply some of the parameters already generated from laboratory experiments to real-world scenarios, improving the reliability and applicability of the research.

The existing research on biochar and its capacity to mitigate heavy metal contamination exhibits encouraging outcomes. Despite progress, there remains a significant knowledge gap regarding the interaction of various heavy metal ions with biochar and its efficacy in industrial contexts. It is essential to conduct experiments at a pilot-scale or industrial level using heavy metal-contaminated effluents to gain a comprehensive understanding of the practical applications of biochar.

## 3 Biochar generation

Biochar is primarily produced through thermal conversion techniques, resulting in alterations to the chemical composition of biomass [30], [85]. Almost all forms of biomass can generate biochar; however, its properties greatly vary based on the biomass used as feedstock, thermal conversion techniques, operation conditions, and types of reactor used for its generation [86], [87], [88], [89]. Generating biochar includes conventional pyrolysis, gasification, and hydrothermal carbonization [90], [91]. It is essential to comprehend the suitability of various conversion technologies with various biomass feedstocks to maximize the utilization of different feedstocks fully. Biochar's ultimate characteristics depend on the feedstock and the respective preparation technique. Therefore, selecting the appropriate feedstock constitutes the initial phase in the biochar preparation process [92]. The production technique can affect the properties of biochar, such as functionality, surface area, and pore size, which can, in turn, affect its adsorption efficiency [76]. Studies have shown that biochar produced through slow pyrolysis can have higher adsorption efficiency than biochar produced through fast pyrolysis or gasification [93]. However, researchers have found that biochar produced through hydrothermal carbonization has a higher surface area and pore volume, which leads to higher adsorption capacity for heavy metals [94], [95].

### 3.1 Conventional pyrolysis

There are three primary types of pyrolysis; slow, fast, and flash - depending on heating rates and solid residency times; this leads to notable distinctions in the amount and properties of solid, liquid, and gaseous products [96]. The solid and liquid products constitute biochar and bio-oil, while the released gases comprise carbon dioxide, carbon monoxide, hydrogen, and syngas (C_1_–C_2_ hydrocarbons) [97]. Slow pyrolysis primarily produces more biochar, whereas bio-oil is the main output for fast and flash pyrolysis [98]. Temperature, heating rate, solid residence duration, particle size, and other parameters appreciably influence the output and characteristics of biochar.

The temperature during pyrolysis is the most crucial factor in controlling biochar output. A low temperature produces a high biochar yield, while a higher temperature substantially reduces the yield [99], [100]. The temperature of pyrolysis affects various properties of biochar, including its carbon structure, functional groups, and various physical and chemical characteristics.

The course of biochar generation takes three fundamental steps: pre-pyrolysis, main-pyrolysis, and carbonaceous product formation. During the first stage, from room temperature to 200 °C, moisture and light volatile compounds evaporate and cause bond breakage and the creation of hydroperoxide, –COOH, and –CO groups [101]. In the second stage, between 200–500 °C, hemicelluloses and cellulose undergo devolatilization and decomposition [102], [97]. Above 500 °C, the final stage involves lignin degradation and other chemically bonded organic matter [103].

As the temperature increases, the carbon structure of biochar's unstable organic carbon, acidic functional groups, and O-alkyl-C decreases, while aryl-C increases due to deoxygenation reactions [104]. This stage leads to increased aromaticity, ash content, pH, hydrophobicity, stability, and degree of crystallinity but decreased biochar yield, hydrophilicity, and amount of oxygen, hydrogen, and sulfur [105], [106], [107]. The ratios of O/C, H/C, (O + N)/C, and (O + N + S)/C also tend to decrease with increasing temperature.

### 3.2 Gasification

Gasification entails a process whereby carbonaceous materials are subjected to elevated temperatures (>500 °C) and limited oxygen to create syngas, tars, and biochar [76], [108]. Biomass gasification typically involves four stages: drying, pyrolysis, partial oxidation, and reduction [109]. The amount and properties of generated products depend on the control and optimization of these four steps.

Yang et al. [110] conducted a comparative study to determine the impact of biochar attributes produced under air-limited conditions (≈7.2 L air, as carried out by Chen and co-workers [111]) on the adsorption performance and mechanism of heavy metals. The biochars were prepared at temperatures ranging from 300 to 750 °C in both nitrogen gas and limited-air environments and then used for the adsorption of cadmium and nickel. They reported that the biochars derived from limited-air pyrolysis exhibited appreciably higher maximum adsorption capacities for Cd(II) and Ni(II) than those derived from inert gas pyrolysis.

In an investigation conducted by Godinho et al. [112], the effectiveness of biochar prepared from co-gasification (air/fuel ratio of 0.2) of a blend of rice husk and corn cob, as well as rice husk and eucalyptus stumps, was evaluated. The two biochars exhibited a relatively low porosity but possessed pronounced alkaline nature, which researchers attributed to a high concentration of mineral content. The authors reported that both types of biochar demonstrated a notable ability to remove Cr(III) ions due to their capability to promote Cr precipitation on the surface. Rohith et al. [113] highlighted that biochar produced from the gasification process consists of alkali and alkaline metals and toxic substances, which include polyaromatic hydrocarbons.

### 3.3 Hydrothermal carbonization

Hydrothermal carbonization (HTC) entails converting biomass into a carbonaceous substance by subjecting the feedstock to high temperature and pressure in the presence of water. During the HTC process, organic matter is heated to around 180–260 °C and subjected to 2–6 MPa pressures for 5–240 minutes in a sealed reactor vessel containing water [76].

Under these conditions, the organic matter undergoes a series of chemical reactions forming hydrochar [114], [115]. The process converts various types of biomass, such as wood, agricultural waste, and sewage sludge, into a carbon-rich carbonaceous material. HTC has several advantages over other biomass conversion technologies, such as pyrolysis and gasification, especially its ability to process a wide range of wet feedstock without pre-drying requirements and produce a high-quality and homogeneous product that is stable and easy to handle [116], [117].

Biochar derived from HTC and other thermochemical conversion processes, such as conventional pyrolysis and gasification, possess distinct physicochemical properties that influence their applicability in various domains. These applications include wastewater remediation, soil improvement, and bioenergy generation [76]. During the HTC process, organic acids are generated, resulting in the formation of hydrochar with acidic properties [118].

The diverse physiochemical properties are due to the differences in the processing parameters of these techniques, which result in varying compositions, structures, and surface areas of the biochars produced. Therefore, understanding the properties of biochar derived from different thermolytic processes is critical for selecting appropriate biochar-based applications and optimizing their performance.

## 4 Biochar modification

**Figure 12:**
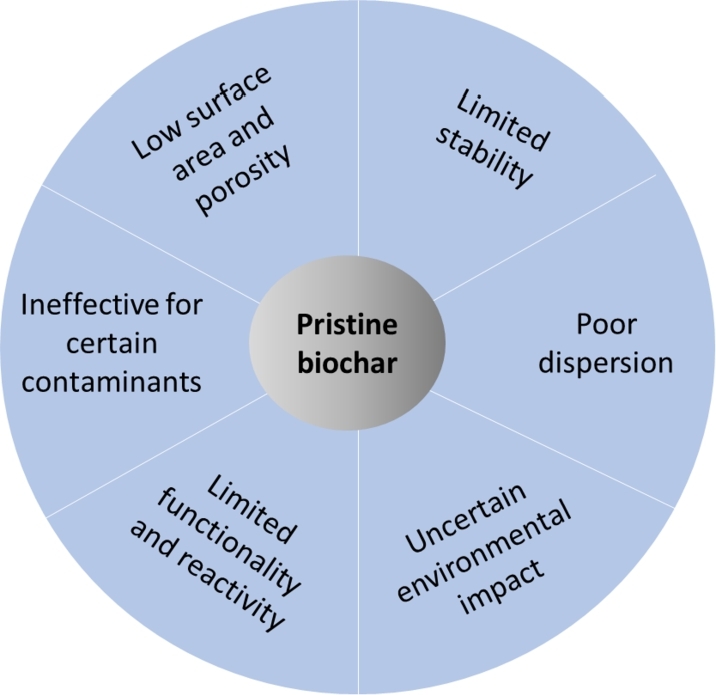
Some of the limitations that may be experienced with unmodified biochar.

The usage of unmodified or pristine biochar usually encounters various limitations. Fig. 12 illustrates some undesirable attributes that may be associated with pristine biochar. Pristine biochar is less effective in removing water pollutants than activated carbon because it has a lower surface area and fewer functionalities. Usually, unmodified biochar has a low specific surface area and pore volume, resulting in low adsorption capacity [119], [120], [121]. The stability of biochar under certain conditions, such as elevated temperature, pressure, and pH, can limit its application [118], [122]. Typically, unmodified biochar exhibits limited effectiveness, but various activation techniques increase surface functional groups, leading to heightened reactivity toward contaminants [121], [123]. It also exhibits being ineffective in the removal of particular contaminants [27], [124], [125].

Generally, biochar displays good dispersion in water [126]; however, in some instances, certain types of biochar can display limited dispersibility for the intended application [127]. Due to its high buoyancy, Liu et al. [128] also drew attention to the formidable challenge of utilizing biochar for cadmium removal. Regardless of the numerous benefits associated with biochar, there are concerns about its long-term potential environmental implications and safety from its usage [129]. Biochar can be prone to physical and chemical degradation, reducing its effectiveness over time and potentially generating undesirable residues that are a safety concern [130], [131]. These factors may influence biochar's utilization and efficiency in removing heavy metal ions from aquatic systems.

There have been numerous efforts to enhance biochar capabilities through modifications to its material characteristics [89]. Biochar can be modified during the preparation techniques specified in Section 3 or after the thermal treatment. Biochar can be modified or activated to enhance its physical and chemical properties, improving its effectiveness for the intended application. Various chemical or physical biochar modifications occur through diverse methods and substances. Such techniques include action treatment with acids, alkalis, salts, metal oxides, ball milling, steam activation, nanoscale zero-valent iron, and other methods [15], [132].

These modifications focus on improving characteristics such as surface area, porosity, and functionality, which ultimately lead to increased adsorption of heavy metals [133], [134]. A widely used modification technique is incorporating substances that can cause the precipitation of heavy metals on biochar [135]. Table 1 summarizes different modifications carried out by researchers to enhance the efficacy of biochar by improving adsorption capacity.

**Table 1:** Comparison of different biochar modifications for heavy metals removal.

No.	Biomass	Biochar	Modification/	Metal ions	Specific surface area			Adsorption capacity		References
	feedstock	preparation:	activation		(m⋅2g^−1^)			(mg⋅g^−1^)		
		pyrolysis (°C)			aPristine	bModified		aPristine	bModified	
1.	Vermicompost	500	KMnO_4_	Cd^2+^	17.07	17.92		78.20	123.69	Zhang et al. [136]
2.	Hickory wood	600	NaOH	Pb^2+^	256.0	873.0		11.2	53.6	Ding et al. [137]
3.	Corn straw	800	B-doped	Fe^2+^	472.06	897.97		(25 °C) 48.62	50.02	Sui et al. [138]
								(40 °C) 66.18	95.09	
4.	Peanut shell	600	cCTAB	Cr^6+^	23.03	14.04		14.56	27.05	Murad et al. [139]
5a.	Cotton straw (JX)	500	BM/FeSO_4_/KMnO_4_	Cd^2+^	14.02	226.50		12.9	65.3	Qu et al. [121]
5b.	Cotton straw (CB)	500	BM/FeSO_4_/KMnO_4_	Cd^2+^	19.13	268.85		16.5	87.2	
5c.	Corn straw (CB)	500	BM/FeSO_4_/KMnO_4_	Cd^2+^	28.54	264.48		22.5	135.2	
5d.	Rice husk (RH)	500	BM/FeSO_4_/KMnO_4_	Cd^2+^	30.35	331.50		20.9	100.9	
6.	Bagasse	450–750	KCl/NaCl	Pb^2+^	294.5	391.9		339	172	Bai et al. [140]
7.	Phoenix tree leaves	500	Fe_3_O4@SiO_2_-NH_2_	Cr^6+^	48.81	56.20		18.2	27.2	Shi et al. [141]
8.	Switchgrass	300HTC	KOH	Cu^2+^, Cd^2+^	2.11	5.01		(Cu^2+^) 4.0	(Cu^2+^) 31.0	Regmi et al. [142]
								(Cd^2+^) 1.5	(Cd^2+^) 34.0	
9.	Banana peel	600	H_3_PO_4_	Mn^7+^, Fe^2+^	11.32	27.41		(Mn^7+^) 0.80	(Mn^7+^) 2.32	Kim et al. [143]
								(Fe^2+^) 27.36	(Cd^2+^) 29.55	
10.	Sewage sludge	500	MgCl_2_	Pb^2+^	40.96	91.57		157.20	887.50	Ngambia et al. [144]
11.	Maize straw	400	–	Hg^2+^, Cd^2+^, Pb^2+^	12.11	–		(Hg^2+^) 173.27	–	Hu et al. [145]
								(Cd^2+^) 22.83	–	
								(Pb^2+^) 81.47	–	
12.	Corn straw	500/800	KOH	Cr^6+^	114.36	2183.79		–	116.97	Qu et al. [146]
13.	Poplar sawdust	300–700	KH_2_PO_4_	Pb^2+^	–	3.01–303.45		24.3	154.7	Xu et al. [147]
14.	Douglas Fir	900–1000	KOH	Cr^6+^, Pb^2+^, Cd^2+^	535	1050		(Cr^6+^)33.5	(Cr^6+^)127.2	Herath et al. [148]
	bark chip wood							(Pb^2+^)84.1	(Pb^2+^)140.0	
								(Cd^2+^)18.0	(Cd^2+^)29.0	
15a.	Plum kernel	500	H_2_SO_4_	Cr^3+^, Pb^2+^	–	146.6		–	(Cr^3+^)14.02	Pap et al. [149]
								–	(Pb^2+^)28.80	
15b.	Apricot kernel	500	H_2_SO_4_	Cr^3+^, Pb^2+^	–	85.6		–	(Cr^3+^)12.69	
								–	(Pb^2+^)23.89	
16.	Kiwi branch	500	Chitosan	Cd^2+^	–	3.30		4.24	118.43	Tan et al. [150]
17.	Molasses	HTC180	FeCl_2_/FeCl_3_/	Pb^2+^, Cu^2+^, Cd^2+^	–	–		–	(Pb^2+^)51.81	Zhang et al. [151]
			Urea						(Cu^2+^)21.41	
									(Cd^2+^)17.64	
18a.	Coffee husk	600	ZnO	As^5+^, Pb^2+^	4.6	3.0		(As^5+^) 3.46	(As^5+^) 4.88	Cruz et al. [152]
								(Pb^2+^) 3.60	(Pb^2+^) 15.00	
18b.	Corncob	600	ZnO	As^5+^, Pb^2+^	24.0	35.0		(As^5+^) 1.99	(As^5+^) 9.62	
								(Pb^2+^) 10.75	(Pb^2+^) 17.00	

^*d*^ BM – Ball-millingBM – Ball-milling

a Pristine biochar

b Modified biochar

c CTAB – Cetyltrimethylammonium bromide (cationic surfactant)

HTC Hydrothermal carbonization

### 4.1 Chemical activation

Chemical activation involves using acids or bases to alter the surface chemistry of biochar, increasing its surface area and reactivity towards contaminants. The chemical activation process can modify the functional groups on the surface of biochar, increasing its ability to adsorb pollutants. Typical acids used for chemical activation include H_2_SO_4_ and H_3_PO_4_ [143], [149], while bases such as NaOH or KOH can also be used [137], [142]. Chemical activation can also introduce new functional groups into the biochar [133], improving its overall performance as an adsorbent. However, it is essential to carefully control the activation conditions to ensure that the biochar remains stable and does not break down over time.

### 4.2 Physical activation

Physical activation involves using heat or pressure to improve the porous structure of biochar [153], increasing its surface area and reactivity towards contaminants [154]. This process increases the porosity of biochar, making it more effective for adsorbing pollutants. Physical activation can be achieved through high-temperature pyrolysis or high-pressure carbonization [30], [155]. These methods can also alter the microstructure of biochar, making it more porous and increasing its overall performance as an adsorbent. Three distinct types of biochar physical activation may include 1) gaseous modification using steam, CO_2_, or air; 2) thermal modification using typical heating; and 3) modification using ultrasonic waves, plasma, and electrochemical techniques [154].

It is paramount to exercise caution when controlling the activation conditions to avoid damaging the biochar and reducing its effectiveness. The specific activation conditions, such as temperature and pressure, can also affect the quality of the final product and its suitability for different water treatment applications. Using chemical and physical modifications to improve biochar properties offers an exciting avenue for sustainable and environmentally friendly applications in various industries, including water treatment, agriculture, and waste management.

### 4.3 Nanoparticles

The enhancement of biochar's removal capacity of heavy metals has been the subject of extensive research, and one of the most promising avenues of investigation has been the modification of biochar with nanoparticles. Studies have demonstrated significant enhancement of biochar adsorption efficiency by incorporating nanoparticles [84], [156]. The resulting biochar-based nanocomposites possess exceptional physical and chemical properties, which arise due to the synergistic effects of both constituents. Tho and co-workers [84] reported a marked improvement in the adsorption capacity for As(III), Cd(II), Pb(II), and Cr(VI) when they modified cassava root husk-derived biochar with ZnO nanoparticles. Their study provides compelling evidence that incorporating ZnO nanoparticles into biochar enhances heavy metals removal capacity. Such findings opened up opportunities for developing highly effective and efficient heavy metal removal strategies based on modified biochar.

### 4.4 Nano zero-valent iron

Nano zero-valent iron (nZVI) has been widely recognized as a highly effective adsorbent for heavy metals [15], [157]. However, the effectiveness of nZVI as an adsorbent for heavy metals is limited due to its small size and susceptibility to oxidation and agglomeration [158]. In order to overcome these limitations, nZVI can be combined with biochar, creating a stable environment that improves the adsorption performance of the composite material. This approach shows promise for improving the efficiency and effectiveness of water treatment applications [159]. Nonetheless, it is essential to note that using nZVI-modified biochar may also release iron ions through leaching into the environment, which can have potential ecological and human health effects. Further studies are needed to understand this approach's benefits and limitations fully.

### 4.5 N-doped biochar

Including nitrogen in the biochar matrix alters its electronic structure, making N-doped biochar more effective at interacting with pollutants such as heavy metals [27]. Doping in biochar modification involves adding small amounts of chemical elements or compounds to the surface. Researchers carry out doping to enhance the properties of biochar for specific applications, such as increasing its adsorption capacity for pollutants like heavy metals [160]. Chemical or physical activation can incorporate the dopants into the biochar. Various nitrogen sources, such as inorganic chemicals (ammonia gas, ammonium salts, and nitric acid), organic chemicals (urea and melamine), and nitrogen-rich biomass (shrimp shell, soybean dreg, and chitosan), have been used to generate N-doped biochars [27], [161].

## 5 Heavy metals removal

### 5.1 Adsorption mechanisms

The effectiveness of biochar in adsorbing heavy metals primarily depends on its surface area, the quantity of active functional groups present on its surface, and cation exchange capacity [162]. The adsorption of heavy metals is a complex phenomenon that involves multiple mechanisms, including physical adsorption, electrostatic attraction, ion exchange, surface complexation, and surface precipitation [15], [125], [163]. Various factors, such as the properties of the adsorbent and the solution, the pH of the system, and the concentration of the metal ions, influence these mechanisms.

#### 5.1.1 Physical adsorption

Physical adsorption, also known as physisorption, occurs when the metal ions are attracted to the surface of the adsorbent through van der Waals forces. Heavy metal adsorption does not depend significantly on physical adsorption as it is typically reversible, temperature-sensitive, and weak [132]. However, some studies have shown that the mobility and availability of specific heavy metal ions can be limited by physical adsorption onto the surface of biochar [164], [165].

#### 5.1.2 Electrostatic attraction

Electrostatic attraction involves the electrostatic attraction between charged adsorbates and adsorbents. The mechanism is highly dependent on ionic strength and the presence of ionic active sites and is often stronger than physical adsorption [166]. In a study by Qiao et al. [167], biochar's physicochemical properties and performance derived from carp residue were evaluated for its efficacy in remediating Cu-polluted water. The authors determined that electrostatic attraction was the most effective potential adsorption mechanism.

#### 5.1.3 Ion exchange

Ion exchange occurs through exchanging of ions between the negatively charged surface groups on biochar and positively charged heavy metal ions. It occurs due to the Coulombic force between the surface groups and ions in the solution. This mechanism is not specific and has a low capacity for adsorption [168]. The mechanism highly depends on the type and concentration of ions on the surface. Wu et al. [169] investigated the efficacy of coconut shell biochar modified with magnesium for removing Pb and Cd from wastewater. The authors attributed the removal mechanism of these heavy metals to ion exchange, among others.

Although modified biochars are highly effective in eliminating heavy metals from aquatic environments, high concentrations of specific metal ions, like Ca, can significantly impede their effectiveness. To address this issue, Lin et al. [170] increased the alkali metal content of biochar to improve its selectivity and enhance its ion exchange capacity. They deciphered that ion exchange was the primary mechanism responsible for the adsorption of Pb and Cd by MgO-modified biochar.

#### 5.1.4 Surface complexation

Surface complexation occurs when metal ions form chemical bonds with functional groups on the adsorbent surface. The mechanism is highly dependent on the nature and concentration of the functional groups. Heavy metal ions can be adsorbed onto the biochar surface by interacting with oxygen-containing functional groups, encompassing hydroxyl, carbonyl, and carboxyl [34]. The oxygen atoms in these functional groups possess lone pairs of electrons that form coordination bonds with the outer orbitals of heavy metal ions. This results in stable complexes that immobilize heavy metal ions [146]. In a study by Tian et al. [171], a magnetic biochar derived from sewage sludge was utilized for the adsorption of Pb^2+^, Cd^2+^, and Cu^2+^. The researchers concluded that surface complexation was one of the mechanisms responsible for the adsorption process.

#### 5.1.5 Surface precipitation

Surface precipitation occurs when metal ions form insoluble compounds on the adsorbent surface. This mechanism highly depends on the system's pH and the metal ions' solubility. Surface precipitation is prone to occur on biochar with a high concentration of phosphate and carbonate ions on its surface. This phenomenon is attributed to the inherent chemical properties of such biochar (e.g., sludge-derived biochar), which facilitate the binding of water molecules to their surface, leading to the formation of precipitation [172].

Understanding these different mechanisms is essential for designing effective biochar composites to remove heavy metals from contaminated water. By manipulating the properties of the adsorbent or the solution, it is possible to optimize the adsorption process and achieve high removal efficiencies.

### 5.2 Factors influencing removal

Various factors can influence the heavy metal ions removal efficiency by biochar, including pH, biochar properties, and metal speciation. The pH of aqueous media can influence the preferential adsorption of particular metal ion species. Uchimiya et al. [173] demonstrated that the addition of biochar increased the pH, which subsequently enhanced the removal of Ni(II) and Cd(II). Varying pH can also affect the oxidation state of the heavy metals, which can, in turn, affect their adsorption on biochar [174].

The pH of biochar, a crucial aspect related to its functional groups, tends to increase with heightened pyrolysis temperature, primarily due to the corresponding increase in ash content [175], [176]. The rise in pH is associated with higher quantities of carbonates and functional groups like COO- and O- [177]. Furthermore, it has been observed that the buffering capacity of biochar varies depending on the source material. Chen et al. [35] highlighted that the application of biochar in simulated acid mine drainage (mine-impacted water) showed remarkable effectiveness in binding metals and neutralizing acidity.

The properties of biochar, such as surface area and pore size, can also affect its adsorption efficiency. Functional groups on biochar surfaces, such as carboxyl, hydroxyl, and phenolic groups, play a significant role in the adsorption of heavy metals [178], [179]. Metal speciation, the different chemical forms of metal, can also affect the adsorption efficiency of biochar, as some forms of heavy metals are more easily adsorbed than others. The presence of other competing ions in the solution can also affect the adsorption efficiency of biochar.

## 6 Spent biochar implications

Adsorption is commonly used in industrial processes and generates a significant amount of spent adsorbents. Disposing these adsorbents, often contaminated with heavy metals, presents a significant environmental challenge as they can cause harm and accumulate in the environment. Current methods of incineration or land-filling exacerbate the solid waste problem and increase the risk of secondary pollution, hindering efforts to achieve carbon neutrality.

The reuse of biochar after heavy metal adsorption is limited, as it may contain high levels of heavy metals and may not be suitable for use as a soil amendment or feedstock for energy production. The safe and effective reuse of used heavy metals-containing biochar has garnered significant interest among researchers and other parties [180], [162].

Two fundamental approaches have emerged for this purpose; stabilization of adsorbed heavy metals and the desorption of adsorbed heavy metals to enable the reuse of biochar are some of the critical approaches to repurpose spent biochar. Different methods for removing heavy metals from spent biochar include chemical, thermal, and microwave-assisted regeneration [181]. The repurpose and regeneration of heavy metal-laden biochars may suffer a drawback because the adsorption capacity and efficiency may need a complete restoration. The biochar eventually becomes waste, an environmental concern, after multiple regeneration cycles.

Stabilization usually involves treating the spent biochar to immobilize the adsorbed heavy metals within its matrix. Developing a stable and efficient method of metal stabilization is essential to ensure the safe application of sewage sludge biochars and minimize environmental risks due to the co-existence of inherent multiple metals and metalloids [182]. The stabilization can occur by incorporating additives that enhance the immobilization of toxic elements during the pyrolysis process, as suggested by Jin and co-workers [183].

A resource utilization strategy can be implemented to reuse the heavy metal-laden spent biochars [184]. Such a strategy aligns with the circular economy principles, which aim to transform waste into valuable and profitable products. Conversion of waste adsorbents into benign substances minimizes environmental impact and has the potential for generating income and creating economic opportunities. The circular economy considers that the outputs of a given process are not regarded as waste but as potential inputs for other processes [185]. As a result, it is possible to use resources more sustainably while reducing the adverse effects on the environment.

Finding effective ways to stabilize heavy metals that have been adsorbed onto spent biochar is still a challenging task. Without proper post-treatment, secondary pollution may occur as environmental conditions change. Yang et al. [186] experimented with stabilizing spent rice straw biochar loaded with cobalt and nickel. They accomplished this through the adsorption of metal ions and then applied hydrothermal carbonization, resulting in a stable layered double hydroxide structure on the biochar surface. The stabilized biochar had a leaching rate below 0.005% and was subsequently reused as a catalyst in the degradation of organic pollutants through the activation of peroxymonosulfate (PMS).

Using a similar approach, Pan et al. [187] utilized spent magnetic biochar (Cu-Fe@BRC) that had Cu^2+^ adsorbed directly onto its surface for the activation of PMS and degradation of norfloxacin (NOR). The study's results revealed a significant improvement in PMS activation and NOR decomposition performance in the Cu-Fe@BRC/PMS system. This outcome was attributed to the enhancement of the surface area and conductivity of Fe@BRC due to the adsorbed Cu^2+^. Furthermore, using density functional theory (DFT) calculations and electrochemical analysis, the authors demonstrated that incorporating Cu^2+^ increased the redox capacity and electron transfer of Fe@BRC. This enhancement in the Fe@BRC's properties is perceived to have facilitated NOR decomposition in the Cu-Fe@BRC/PMS system.

To establish the reusability of spent biochar, some researchers have made supercapacitor electrodes using spent biochar loaded with heavy metals [188], [189]. The escalating demand for green and clean energy drives the development of affordable and effective electrode materials for supercapacitors. Yao and Wu [189] highlighted that biomass-derived carbon materials have a high capacity for removing heavy and toxic metal ions from wastewater, resulting in a porous carbon material with adsorbed metal ions. Therefore, the similarities in structure between air cathodes and the metal-adsorbed carbon biochar can effectively link these two areas.

Biochar with a well-structured composition is highly desirable as a material for supercapacitor electrodes. Chang et al. [190] used cobalt to guide the graphitization process and enhance the electrochemical performance of biochar. The resulting cobalt-doped biochar exhibited remarkable electrochemical properties, making it a promising candidate for various energy storage purposes.

Conversion of heavy metal-laden spent biochar into effective electrocatalysts could aid in tackling the issue of increasing energy demand. Chen et al. [191] demonstrated that spent adsorbents can be converted into heterostructured electrocatalysts through a simple boriding process. They pointed out that the spent adsorbents containing metal ions in the biochar were converted entirely into heterostructures of magnetic metal borides and biochar. These new structures showed good performance in the oxygen evolution reaction.

The advanced oxidation process (AOP), a highly effective method for removing organic pollutants, relies on the generation of hydroxyl radicals (⋅OH). Mer et al. [192] applied biochar loaded with lead and nickel to catalyze the in-situ generation of the highly reactive ⋅OH radicals. Their findings indicate that the efficacy of the AOP process depends on the structure of the carbon material and the identity and quantity of the metal complexes within the material.

These examples highlight the approaches used to mitigate the risk of secondary pollution by preventing the desorption of heavy metal ions from spent biochar while promoting novel and optimal utilization of biochar for environmental remediation.

## 7 Future research direction

### 7.1 Biochar composites

Researchers have been exploring methods to enhance the adsorption capacity of biochar, as highlighted in Table 1. Further research is required to develop biochar formulations tailored explicitly for removing specific heavy metals. This research endeavor could involve incorporating functional groups or using specific feedstocks to improve the adsorption capacity for certain metals.

The bibliometric analysis highlighted the prevalent inclusion of nanoparticles, nanocomposites, or nano zero-valent iron to prepare biochar composites. Highlighting the effectiveness of biochar for heavy metal remediation can be further enhanced by incorporating novel substances to improve biochar's physical or chemical properties. The aspiration lies in increasing adsorption capacity and improving selectivity for particular heavy metal ions.

Utilizing novel materials and innovative techniques can significantly enhance biochar's efficacy toward removing heavy metal species in wastewater treatment applications. Further research and development on these materials have the potential to produce novel biochar composites for effective remediation of heavy metal-contaminated water.

### 7.2 Acid mine drainage

Based on the trending topics identified by the conducted bibliometric analysis, the researchers are exploring the efficacy of biochar in treating AMD. While promising results have been reported in the literature, further investigation is needed to comprehend its effectiveness better and optimize its application. Using modified biochar in conjunction with other treatment methods, such as phytoremediation and microbial treatment, may yield improved results in heavy metal removal with minimal environmental impact. Therefore, it is recommended that more attention be devoted to this area of study to develop a more comprehensive understanding of biochar's potential benefits in treating acid mine drainage.

### 7.3 Reusability of spent biochar

Safe and effective reuse of spent biochar that contains heavy metals is an area of active research and development. The stabilization and desorption of adsorbed heavy metals are two viable approaches for this purpose, and their continued exploration and refinement hold promise for the development of sustainable solutions for the treatment and management of heavy metal-contaminated wastes.

### 7.4 Modeling by artificial neural networks

The complex nature of the adsorption process makes it challenging to model and predict biochar-based adsorbents' performance accurately. Some researchers have embarked on using artificial intelligence (AI) methods, such as artificial neural networks (ANNs), in the modeling of heavy metal adsorption by biochar [193], [194]. The success of these models can be used to predict the performance of biochar-based adsorbents under different conditions, such as changes in pH, temperature, and concentration of heavy metals in the wastewater.

Sivamani et al. [195] employed a Feed-Forward Back-Propagation Neural Network (FFBPNN) and Box-Behnken Design (BBD) model to predict the adsorption of Cu(II) by biochar made from orange zest. The findings displayed a good correlation between the experimental data and the predicted values from the BBD and FFBPNN models.

A study by Zheng and Nguyen [196] utilized a cutting-edge artificial intelligent model to predict heavy metal removal efficiency from aqueous solutions using biochar. The study involved the utilization of 44 biochars and 353 experimental works aimed at removing six different heavy metal ions (Cu^2+^, Pb^2+^, Zn^2+^, As^3+^, Cd^2+^, and Ni^2+^) from water. To optimize the parameters of the developed artificial neural network (ANN) model, the researchers employed the queuing search algorithm (QSA) in conjunction with the ANN model. The study's findings revealed that the proposed optimization QSA-ANN model outperformed the traditional ANN model in accuracy.

These studies showcase the potential of harnessing advanced technologies to enhance the efficiency and accuracy of environmental remediation processes. The use of AI techniques such as ANNs in modeling heavy metals removal by biochar represents an exciting area of research that can significantly improve insights into the adsorption process and the performance of biochar-based adsorbents.

### 7.5 Sustainable energy solutions

The conversion of exhausted biochar into electrode materials for supercapacitors and catalysts aligns with the drive for sustainable energy solutions. The study field emphasizes the significance of carbon nanostructure in creating advanced energy storage materials. It paves the way for further exploration in this area [184], encompassing the reprocessing of heavy metal-laden spent biochars. Despite using different carbon materials derived from biomass waste for supercapacitor electrodes, there still needs to be more knowledge on the connection between the precursor of biomass waste and the resulting carbonaceous electrode materials.

## 8 Conclusion

The application of science mapping in this study has provided a comprehensive understanding of global trends and patterns in using biochar for heavy metals remediation. The results show that China, the United States, and India have assumed prominent roles in this field, and the usage of biochar is gaining momentum. The study also revealed that chromium, lead, cadmium, and copper are the most researched heavy metals in this area, while rice husk, rice straw, and sewage sludge are the most utilized feedstocks. The formulation of innovative biochar composites represents a promising avenue for achieving effective remediation of heavy metals. By harnessing the unique properties of biochar, combined with carefully selected additives and processing techniques, these composites have the potential to provide a highly efficient and sustainable solution for mitigating the harmful effects of heavy metal contamination in a variety of settings. Through ongoing research and development efforts, the utilization of biochar to remediate heavy metals in aquatic environments continues to evolve, paving the way for increasingly sophisticated and effective strategies for addressing this critical environmental challenge. Looking ahead, the future of using biochar in heavy metals reduction is compelling, with the anticipation of various innovative configurations of biochar composites, spent biochar being utilized in supercapacitors and catalysts, and the testing of AI models such as ANN to simulate experiments. This study provides researchers, policymakers, and stakeholders valuable insights to develop effective biochar heavy metals remediation strategies.

## CRediT authorship contribution statement

**Zebron Phiri:** Conceptualization, Data curation, Formal analysis, Investigation, Methodology, Validation, Visualization, Writing – original draft, Writing – review & editing, Software. **Nathaniel T. Moja:** Data curation, Validation, Visualization, Writing – review & editing. **Thabo T.I. Nkambule:** Funding acquisition, Project administration, Resources, Writing – review & editing. **Lueta-Ann de Kock:** Funding acquisition, Project administration, Resources, Supervision, Writing – review & editing.

## Declaration of Competing Interest

The authors declare that they have no known competing financial interests or personal relationships that could have appeared to influence the work reported in this paper.

## Data Availability

The additional data supporting this study's findings are available in the supplementary material deposited in Mendeley Data with the identifier doi:10.17632/yyff8sz32t.1.
